# Social Infrastructure and Street Networks as Critical Infrastructure for Aging Friendly Community Design: Mediating the Effect of Physical Activity

**DOI:** 10.3390/ijerph191911842

**Published:** 2022-09-20

**Authors:** Jiayi Jiang, Zhengwei Xia, Xiaodi Sun, Xuanxuan Wang, Shixian Luo

**Affiliations:** 1 School of Architecture, Soochow University, No. 199 Ren-ai Road, Suzhou Industrial Park, Suzhou 215123, China; 2 Department of Environmental Sciences and Landscape Architecture, Graduate School of Horticulture, Chiba University-Matsudo Campus, Chiba 271-8510, Japan

**Keywords:** older adults, physical activity, community social infrastructure, community street networks, age-friendly environment, healthy aging

## Abstract

Establishing an age-friendly environment at the community level is essential for promoting healthy aging. This study focused on the relationship between older adults and the community environment through their levels of satisfaction within it. We measured their physical activity (PA) in the community environment and three variables of community-level satisfaction: community environment (SCE), community social infrastructure (SSI), and community street networks (SSN). We analyzed 108 older adult participants in Suzhou using mediation analysis and multiple linear regression to investigate the relationship between physical activity and the community environment. The results of the mediation effect model showed that SCE, SSI, and SSN all affected the physical functions of older adults via the total amount of physical activity (TPA); SSI and SSN affected older adults’ physical functions by affecting the total duration of moderate-intensity physical activity (MPA) and vigorous-intensity physical activity (VPA). In addition, SSI and SSN are related to the types of community facilities, street space quality, and accessibility. Our study provides valuable insights into optimizing aging-friendly neighborhoods through moderate-to-vigorous-intensity PAs at both the facility and street space levels.

## 1. Introduction

The global population is aging rapidly. In 2019, the number of people aged 60 years and older was 1 billion (13%), and this number is expected to increase to 2.1 billion by 2050 (22%) [1]. Within the next few decades, one of the biggest challenges for public health will be increasing healthy aging via the maximization of health-adjusted life expectancy (HALE) [1]. Evidence from around the world has shown that health-related risk factors at the community level extend beyond the individual [2], and this is even more evident among older adults [3]. Older adults prefer to spend much of their time within their homes and community. Therefore, there will likely be great utility in maintaining and enhancing community sustainability and promoting healthy communities. Local-level community environmental factors, particularly neighborhood walkability [4], recreational facilities [5], and green space [6], increasingly provide a focal point for possible interventions that could be used to realize the goal of healthy aging.

Numerous studies in public health, urban planning, and environmental psychology have demonstrated the correlation of older adults’ physical and mental health with various aspects of the physical and social environment. One effective way to promote physical and social environments for older adults’ health is through physical activity (PA). PA can slow disease progression in older adults, improve their functioning in daily life, and promote healthy aging [7]. Moreover, these studies proved that PA participation is an essential mediator of the community environment to promote older adults’ daily living abilities [8]. Despite the numerous benefits of PA, it is difficult for most older adults to achieve a sufficient amount of PA primarily due to time constraints, lack of local facilities to perform PA, and daily living abilities [9]. Past research has provided data linking physical activity (PA), local facilities, and older adults’ daily living abilities [9]. These studies have established independent connections between community environment and PA participation. Some studies have shown that the built environment further affects older adults’ health by promoting or hindering PA [10,11]. Other studies have focused on differences in the effects of the community living environment and the socioeconomic attributes related to PA in older adults [12].

However, community environments’ influence on older adults’ PA remains less explored according to the intensities of PA [12], which is a significant knowledge gap since there is a close association between PA intensities and living ability among older adults. The World Health Organization (WHO) guidelines on PA and sedentary behavior state that moderate-intensity physical activity (MPA) and vigorous-intensity physical activity (VPA) are essential in maintaining physical and mental health in older adults [13]. Moreover, compared with light-intensity physical activity (LPA), moderate- and vigorous-intensity PA duration in older adults is seriously insufficient. Nevertheless, few researchers have addressed how PA intensity correlates with the community environment and analyzed their association with physical functioning in older adults.

At the same time, physical spaces and facilities and the social environment provide people with opportunities to participate in PA and socialize with each other, which in turn affect their physical and mental health. Different dimensions of built environment characteristics are related to varying intensities of PA. The availability, quality, accessibility of relevant facilities, infrastructures, and spaces, and the safety conditions of the community environment can influence the intensity, direct or indirect, duration, and frequency of physical activities and hence indirectly affect older adults’ living ability. Therefore, dimensions of built environments include community design, public transport networks, parks, and other social infrastructure [14]. The elements, including facility quality, style, environmental quality, greenery, walkability, safety, and street connectivity, are positively or negatively associated with PA in older adults for different activity-related purposes and intensities [15]. For instance, neighborhood walkability positively correlates with total PA in older adults. Furthermore, walking is the most popular light-intensity physical activity (LPA) among older adults, and this activity is primarily related to community street networks (CSN) [5]. Increasingly, evidence suggests that designing CSN for greater compactness, connectivity, and improved configurations can improve residents’ physical activity levels and health outcomes [16]. In addition, recent research has considered poor social infrastructure (SI) to be a critical factor in exacerbating gender and age disparities as well as undermining health and wellbeing [17,18]. Overall, the effects of built environments, especially social infrastructures, and public street networks, were critical to older adults’ collaborative economy, productivity, and quality and cost of living [19].

Past studies have also shown that the availability of local facilities can increase satisfaction within the community environment and the self-rated health of older people [20]. Moreover, older adults’ satisfaction significantly impacts their sense of community and mental health [21]. Therefore, we used older adults’ self-reported satisfaction levels regarding CE, SI, and SN to determine the overall quality of their community environment. To fill the knowledge gap mentioned above, we have developed a new survey-based way to measure community environmental features and their potential impacts on health-related outcomes through different intensity PA concerning healthy aging among older adults. Therefore, in this paper, we focus on the two main environmental factors, social infrastructure, and street space, to analyze how they affect older adults’ physical functions through different intensities of PA. The research objectives were as follows:

(1) explore the community characteristics that affect older adults’ functional capability by influencing the three different intensities of PA.
(2) understand the environmental factors affecting older adults’ satisfaction levels with their community environment.

## 2. Materials and Methods

### 2.1. Conceptual Framework

The person-environment fit theory raised by Lawton provides a useful framework for understanding the range of social and physical environmental factors that influence older adults’ health and well-being [22]. Moreover, many survey instruments have been developed to assess community environment factors for their impacts on older people’s health and daily activities. Table 1 shows the main tools and measures for understanding and optimization of the associations between older adults and the surrounding social and physical environmental context at a community level. In addition, the existing survey instruments from the conceptual frameworks of environmental gerontology [23], ecological theory of aging [24], and social-ecological model of health promotion [25] provide valuable insights to support our study of the mediating effect of PA on older adults living abilities. Guided by these theories, the final measurement dimensions are composed of dimensions of built environment characteristics (e.g., safety, street walkability, and social infrastructure), health behaviors (LPA, MPA, and VPA), and health outcomes (physical functional capability, which is measured via the IADL scale).

### 2.2. Study Samples

**Figure 1:**
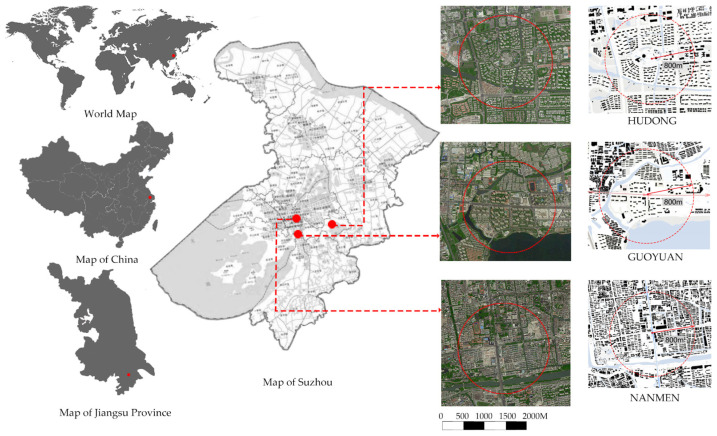
Study site. The study area is a circular area with a diameter of 800m.

Our study area is in Suzhou, also called Soochow, situated on the southern section of the Grand Canal on a generally flat, low-lying plain between Lake Tai to the west and Shanghai to the east [34]. The present study was conducted on 142 older adults aged 65 and over between 25 August and 15 September 2020 who had resided in downtown Suzhou for more than three years. In order to ensure the consistency of the research results, we limited the scope of the study area (less than 5 degrees) to avoid the influence of walking resistance on the research results. Figure 1 shows the study site. As research cases, we selected three representative communities in Suzhou City, including Nanmen, Guoxiang, and Huodong. Nanmen is a typical traditional residential community in the old city, Guoxiang is a modern residential community model, and Hudong is a community representative of the neighborhood center model in Singapore. We investigated older adults within 800 m of the subway station. The studied activities were directly related to the place of residence within 1000 m walking distance and about 15 min from their home.

The research was supported by JUWEIHUI (local neighborhood committees) and conducted via field research. All the subjects of this study were older adults without visual impairment. After excluding 34 participants who responded insincerely, the data of 108 remaining participants were subsequently analyzed.

### 2.3. Data Collection

#### 2.3.1. Physical Activity (Mediating Variable)

The PAs of older adults were test determined on four levels: the total amount of physical activity (TPA) per week, LPA, MPA, and VPA. These were determined via a self-reported questionnaire. LPA includes walking, playing most instruments. MPA includes bicycling with light effort and heavy cleaning. VPA includes running, jogging, singles tennis, bicycling fast, and playing ball games. Participants were asked to report their average duration and frequency of these three intensities of PA over the previous week. We calculated the total duration of PA for each intensity. TPA is the weighted total time of the three intensities of PA.

#### 2.3.2. Physical Functional Capability among Older Adults

We used functional capability to measure older adults’ physical health. The Lawton instrumental activities of daily living (IADL), developed by Lawton and Brody (1969) [22], were used to measure the functional capabilities of older adults. The Lawton IADL includes seven items, use of the telephone, traveling via car or public transportation, shopping, meal preparation, housework, medication use, and financial capacity, and each criterion is graded on a three-point scale: independent, assistance needed, or dependent (1, 2, 3) [35]. The score of functional capability among older adults was from 0 to 21.

#### 2.3.3. Dimensions of Built Environment Characteristics

This study examined the dimensions of built environment characteristics for three categories: community social infrastructure (SI), community street networks (SN), and the overall community environment (CE). We investigated the subjective satisfaction of older adults with their community environment (SCE), social infrastructure (SSI), and street networks (SSN), respectively. We divided subjective satisfaction into a score of 1–5 with very satisfied (5), slightly satisfied (4), neutral (3), slightly unsatisfied (2), and very unsatisfied (1). Social infrastructure (SI) was divided into educational infrastructure (EDU), health and aged care services (HCS), commercial services (CS), arts and cultural infrastructure (ACI), green and blue space (GBS), and recreational infrastructure (RI) [36]. Referring to the research structure developed by Mehta [37] and Alfonzo [38], we examined community street networks (SSN) across four dimensions: communication, feasibility, amenity, and safety [39]. The satisfaction elicited by community street networks (SSN) was measured via public communication spaces (PCS), green space (GS) [40], street accessibility (SA) [41], street walkability (SW) [42], the diversity of resting facilities (DRF) [43], and the separation of people and vehicles (SPV). Table 2 shows the contents of the questionnaire.

#### 2.3.4. Socioeconomic Attributes (Covariant)

Six socioeconomic attribute variables (age, gender, household income, occupation, education level, and household structure) were included as the covariants. Gender was classified into “male” and “female”; household income was classified into four levels (“under 3000 RMB”, “3000 to 5000 RMB”, “5000 to 10,000 RMB”, and “over 10,000 RMB”); education level was divided into “junior high school or below”, “high school”, and “college or above” [44]. For family characteristic variables, we classified the household structure into “lives alone”, “living with a spouse”, “living with children”, and “living without children” [45].

### 2.4. Statistical Analysis

The original data were filtered prior to conducting statistical analysis. SPSS software (version 25.0 for Windows; SPSS, Chicago, IL, USA) was used for descriptive statistics and correlation analysis. The Hayes PROCESS macro (Model 4) was used to test the hypotheses in this study [46].

In part 1 of the statistical analysis, we used mediation analysis to examine whether PA (LPA, MPA, VPA, and TPA) mediated the relationship of the community environment (measured via SSI, SSN, and SCE, separately) with older adults’ physical functions (measured via the IADL scale), with age, gender, income, occupation, and education level as the covariables. Finally, a 95% percentile bootstrap confidence interval (CI) with 5000 bootstrapping samples was used to determine and quantify the statistical significance of the model. The indirect effects were deemed significant if 0 was not included in the bootstrap confidence intervals [47].

In part 2 of the statistical analysis, we used stepwise linear regression analysis to evaluate the exact built environment characteristics that were associated with residents’ satisfaction with the community environment. This method allowed the independent variables to be gradually entered and removed based on improvement by at least 1% in the adjusted coefficient of determination (adjusted R^2^). Additionally, the independent variables with negligible t-statistics at 95% confidence level (*p* > 0.01) were removed. Finally, the model coefficients were reported.

## 3. Results

### 3.1. Descriptive Statistics

A total of 108 older adults were included in the study. Table 3 describes the characteristics of the participants with regards age, gender, education level, household income, household structure, and occupation.

### 3.2. Results of Mediating Effect Model

**Figure 2:**
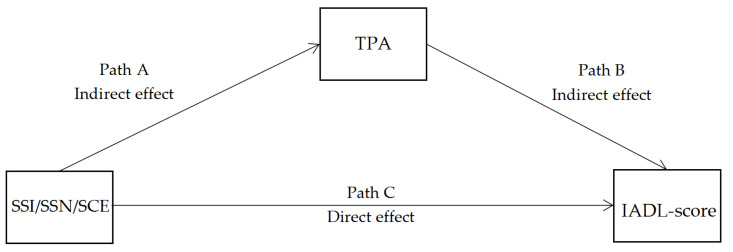
The mediating effect model.

**Figure 3:**
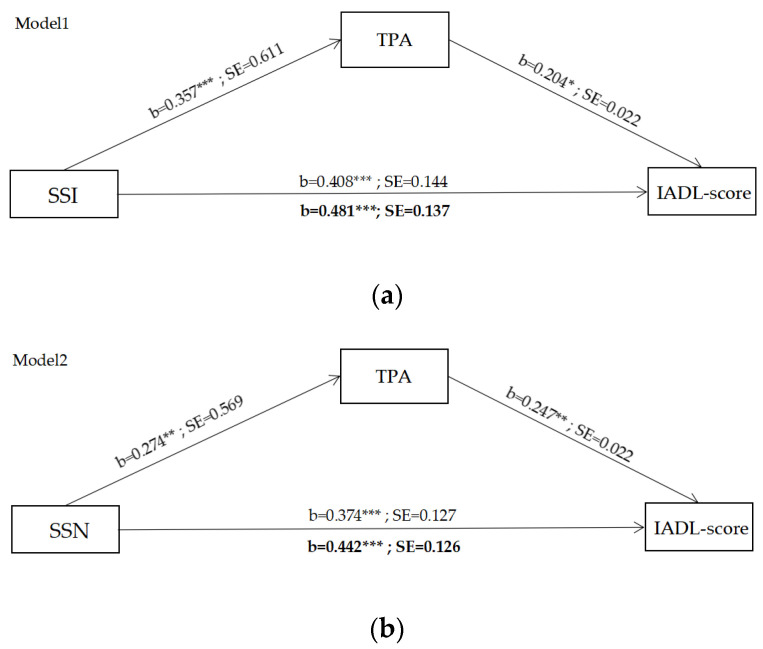

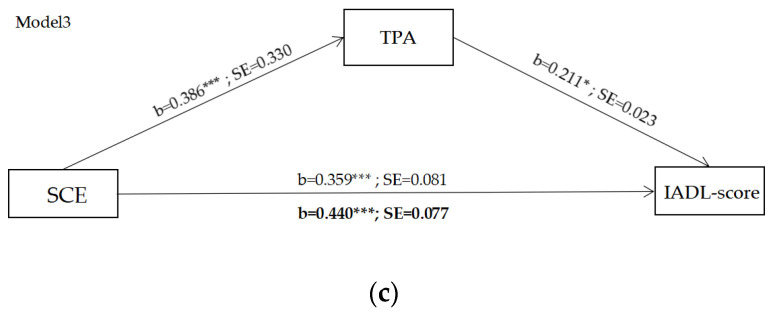
Results of the mediating effect model: (**a**) model 1, the effect of SSI to IADL score mediated by TPA; (**b**) model 2: the effect of SSN to IADL score mediated by TPA; (**c**) model 3: the effect of SCE to IADL score mediated by TPA (direct effect of X on Y in boldface). * *p* < 0.05, ** *p* < 0.01, *** *p* < 0.001.

Figure 2 shows the mediating effect model. Figure 3a–Figure 3c shows the mediating effects of TPA on older adults’ physical functions (measured via the IADL scale). Figure 1 also presents the lower limit confidence interval (LLCI), the upper limit confidence interval (ULCI), and the probability test value (*p* value) to evaluate the significance of these mediating effect models. The DW value of each mediating effect model is close to 2.0, indicating no multicollinearity among the variables. Table 4 depicts the indirect effect effects of TPA on older adults’ physical functions. The bootstrap-derived 95% confidence intervals do not include zero for any outcomes of models 1, 2, and 3. Therefore, there were significant indirect effects of SSI, SSN, and SCE through TPA on all physical functions of older adults (IADL score), specifically, SSI (coefficient = 0.111, 95%BootCI (0.009, 0.285)), SSN (coefficient = 0.094, 95%BootCI (0.002, 0.251)), and community satisfaction (SCE) (coefficient = 0.068, 95%BootCI (0.004, 0.175)). The indirect effect of all models was positive because path A and path B were both positive, thereby suggesting that high values for SSI, SSN, and SCE could increase TPA, which consequently improves older adults’ physical functions.

**Figure 4:**
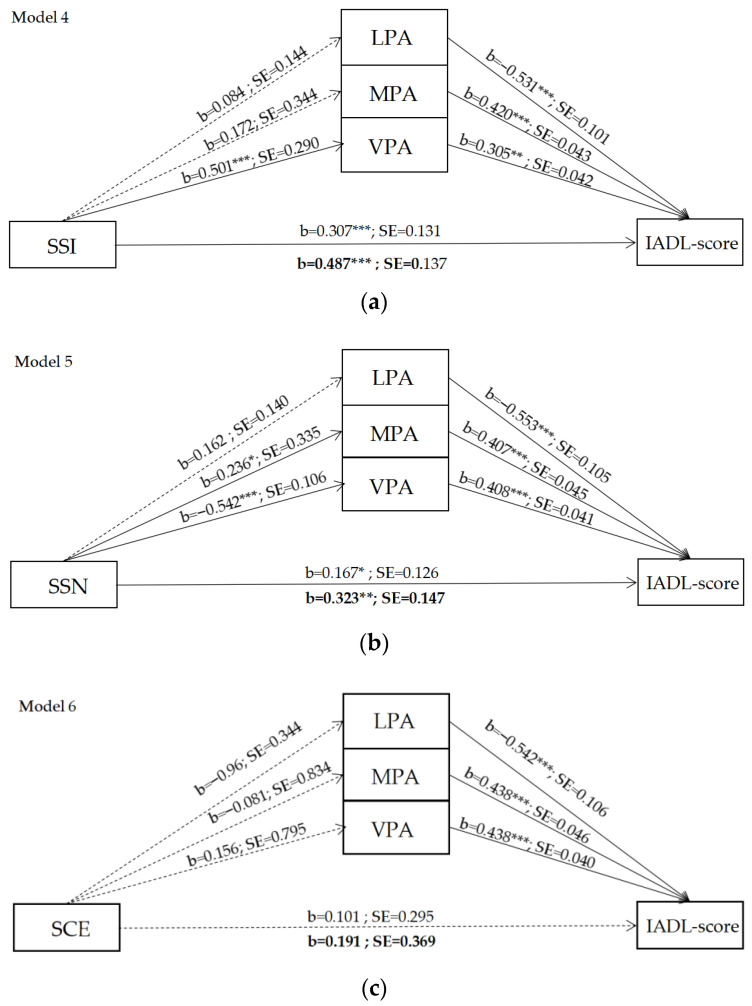
Results of mediating effect model: (**a**) model 4: the effect of SSI to IADL score mediated by LPA, MPA, and VPA; (**b**) model 5: the effect of SSN to IADL score mediated by LPA, MPA, and VPA; (**c**) model 6: the effect of SCE to IADL score mediated by LPA, MPA, and VPA (direct effect of X on Y in boldface). * *p* < 0.05, ** *p* < 0.01, *** *p* < 0.001.

Figure 4a–c shows the effect of the community environment factors on the IADL-score mediated by LPA, MPA, and VPA. Referring to model 4, we observed that SSI increased older adults’ time spent in VPA, thereby increasing older adults’ physical functions and IADL score. Table 5 depicts the indirect effect of TPA on older adults’ physical functions. Although the indirect effect through LPA and MPA did not significantly impact the IADL score, the total indirect effect was significantly associated with the IADL score (coefficient = 0.276, 95%BootCI (0.098, 0.480)). In model 5, we observed that SSN increased older adults’ MPA (coefficient = 0.146, 95%BootCI (0.014, 0.342)) and VPA (coefficient = 0.227, 95%BootCI (0.113, 0.376)), thereby increasing older adults’ physical functions and IADL score. Although the indirect effect through LPA did not significantly impact the IADL score, the total indirect effect was significantly associated with the IADL score (coefficient = 0.237, 95%BootCI (0.074, 0.438)). Referring to model 6, we observed that there was no indirect effect through PA on the IADL score.

### 3.3. Results of the Multilevel Regression Model

#### 3.3.1. Influences of Community Facilities on Older Adults’ SSI

Table 6 depicts the significant impact of community facilities on SSI. For the MLR model, only four out of the six predictors entered the forward stepwise regression. The model for SSI had an R^2^ = 0.714 and F = 56.638 (*p* < 0.001), thereby explaining 71.4% of the influence mechanism of different types of community facilities on older adults’ satisfaction with community facilities. The coefficients of ACI, GS, and CS positively impacted older adults’ satisfaction with social infrastructure (SSI) with a B of 0.328, 0.329, and 0.170. In contrast, education infrastructure (EDU) was negatively related to older adults’ SSI (−0.746).

#### 3.3.2. Influences of Street Network Quality on Older Adults’ SSN

Table 7 depicts the significant impact of street network quality on SSN. For the MLR model, only four out of the six predictors entered the forward stepwise regression. The model for SSI had an R2 = 0.650 and F = 43.550 (*p* < 0.001), thereby explaining 65.0% of the influence mechanism of street network quality on older adults’ satisfaction with community street networks. All variables, such as PCS, DRF, SPV, and GS, were positively related to SSN with a B of 0.291, 0.235, 0.230, and 0.177.

## 4. Discussion

To our knowledge, this study is the first attempt to differentiate PA intensity to understand the mechanisms of community setting health promotion in older adults. There is strong evidence that the neighborhood-level built environment affects older adults’ physical functions via three mechanisms. Firstly, after adjusting for potential confounders, we observed that subjects’ satisfaction with community social infrastructure (SSI), street networks (SSN), and the community environment (SCE) increased the total time they spent engaging in PA (TPA) and further promoted their living ability. Secondly, we observed that SSI and SSN both promoted older adults’ physical functions by promoting the total time older adults spent in moderate-intensity physical activity (MPA) and vigorous-intensity physical activity (VPA). Finally, community facility types and street network quality were strongly related to older adults’ SSI and SSN, which provides valuable insights into local level planning to maximize healthy aging.

### 4.1. The Effects of SSI, SSN, and SCE on Physical Functions Mediated by PA

The underlying mechanisms linking the community environment and older adults’ physical functions can be explained completely by TPA. Overall, the results indicated that those who reported a higher degree of satisfaction with social infrastructure (SI), street networks (SN), and the community environment (CE) were more likely to engage in physical activity, which could increase these older adults’ physical function. Our findings are consistent with results from previous studies showing that older adults tend to be more physically active in neighborhoods that elicit a higher level of satisfaction with the community environment [48,49,50]. Compared with specific community facilities and older adults’ satisfaction with better street networks, the overall community environment significantly influenced their TPA. A possible explanation for this result is that community members who experience a higher level of satisfaction not only meet their needs for PA but also engage in supportive social networks, which further encourage all types of PA [51]. Evidence shows that community with a higher satisfaction and features promoting intergenerational and peer interactions can help reduce ageism, loneliness, and social isolation as well promote physical activity and health among older adults [51].

These results further support the need to distinguish different intensities of physical activity. Consistent with our expectations, we found that the mediating effects of PA on the impacts of SSI and SSN on older adults’ IADL scores were related to the intensity of PA. Firstly, we observed that for the impact of SSN on older adults’ IADL scores, the greater mediating effect was for VPA, while the smaller was for MPA. Interestingly, although experience shows that LPA is the type of PA with the highest frequency and duration in older adults [52], the role of the community environment in promoting the living ability of the elderly through LPA is not significant. The results may be because, for older adults with self-care ability, LPA is a part of their daily life (e.g., grocery shopping, walking) and has little correlation with the quality of the community environment. Secondly, VPA represented the only practical mediating effect of SSI on older adults’ IADL scores. One possible explanation for this result might be that community street networks are essential places for older adults to conduct VPA such as running, jogging, and fast bicycling. Past studies highlight that most young and middle-aged to old adults underestimate the intensity of PA that is required to achieve health benefits [53]. Our findings provide useful insights into improving VPA in older adults at the community level. It is worth noting, however, that older adults’ satisfaction with the overall community environment was not associated with any particular intensity of PA, only TPA. This may be because the overall satisfaction with the community environment reflects multiple aspects, such as social networking and the physical environment, thereby rendering it difficult to clarify its relationship with different PA intensities. Therefore, it is necessary to distinguish different environment types and further analyze environmental factors related to older adults’ physical health and different PA intensities.

### 4.2. The Effect of Environmental Factors on Older Adults’ Satisfaction Levels

Eight environmental attributes were significantly linked with the satisfaction of SI and SN among older adults in the neighborhood. Specifically, consistent with findings reported from previous studies, green space (GS) exerted a positive impact on older adults’ reported satisfaction with SI and SN. Moreover, the positive of GS on SSI is more evident than that for SSN. Compared with street green space (GS of SN), dedicated community parks (GS of SI) have a more significant positive effect on promoting MVPA among older adults. This may be because dedicated community parks provide more decadent resting spaces and more places to rest. These findings are consistent with earlier reports. GS promotes older adults’ life satisfaction by mitigating the negative effect of urban density [54], air pollution and thermal stress [55], depression [56], and other conditions that are common among older adults [43]. However, recent studies depict that communities with more parks nearby might result in a steeper decline in MVPA levels over time [57], which may be verified in our future longitudinal studies. There was only one physical environmental variable (education infrastructure, EDU) that showed a negative association with SSI, which might be related to older adults’ family structures. Communities with a higher proportion of EDU are more likely to attract families with children, thereby resulting in a higher proportion of older adults traveling with children [58]. Arts and cultural infrastructure (ACI) is another positive factor related to SSI, which might be explained by older adults’ higher cultural-related leisure needs [59]. Commercial services were also significantly associated with SSI, and this might be related to the lower occurrence of online shopping among older adults [60]. Moreover, residents’ daily travel to local stores is a major indicator of health, especially for older adults [61].

Public communication spaces (PCS) and the diversity of rest facilities (DRF) were the two most significant factors found to influence SSI. This indicates that socially supportive relationships might impact older adults’ satisfaction levels [5]. For example, community street spaces could serve as places for active travel and social interaction among older adults [62]. Therefore, these could promote older adults’ physical activity and mental health. The physical separation of people and vehicles (SPV) also promoted older adults’ SSN. Recent studies have provided some valuable insights into older adults’ cycling behavior. Some older adults expressed concerns about traffic safety problems and environmental factors such as the separation of people and vehicles, clear signage and markings, and spacious streets [63].

In conclusion, the community environment appears to exert considerable impacts on older adults’ satisfaction with their local neighborhood, reflecting not only their PA and physical functions, but also the attitude of older residents towards community safety, facility availability, and their experiences of using the local amenities and services [64].

### 4.3. Planning Implications

This research provides new insights into the renewal of community facilities, streets, and environments in supporting healthy aging. Referring to our findings, we argue that three aspects should be highlighted for building healthy aging communities:

1. Pay more attention to the impact of community streets on vigorous-intensity physical activity (VPA) of older adults. Provide supportive social relationships through age-friendly street networks and, specifically, focus on street green space quality and street safety.
2. Provide supportive social relationships through age-friendly infrastructures, especially public communication spaces and the diversity of rest facilities.
3. Provide full coverage and high-quality community facilities, primarily cultural, artistic, and commercial services.

### 4.4. Limitations of the Study

Several limitations of the current study are noted: (1) This research only focused on the subjective feelings of residents regarding their local physical environment and therefore lacked objective quantification. (2) The research is limited by the willingness of older adults to accept interviews, our sample size was relatively small, and we only considered the older adults group with self-care ability. We would include more samples and conduct comparative studies to improve the experimental results in the future study. (3) Our study did not consider more detailed facility elements such as public toilets and the number of seats, and we would include them in future research. (4) Despite the availability of valid psychometric scales, all measurements were self-reported or self-evaluated, thereby allowing for relatively more reporting bias compared to more clinically-based data. (5) There exists some discrepancy between the subjective and objective ages that characterize older adults, and cognitive differences at the individual level were not considered.

## 5. Conclusions

Older adults tend to engage in PA more while residing in an enjoyable environment, thereby leading to better physical functions. Therefore, the design and management of the community environment are both essential for creating a healthy aging community. This study surveyed 108 seniors in three communities in Suzhou through questionnaires, whereby their PA conducted within the community was reported alongside three variables of community-level satisfaction (community environment, community social infrastructure, and community street networks). Subsequently, the relationship between these variables was explored using mediation analysis and regression analysis. The main results were as follows:

1. Three variables of community-level satisfaction (the overall community environment, street networks, and social infrastructures) affected older adults’ physical functions by influencing older adults’ TPA.
2. Two variables of community-level satisfaction (SSI and SSN) affected older adults’ physical functions by affecting the total duration of MPA and VPA, especially VPA.
3. GS and ACI positively correlated with SSN, PCS, and DRF, while SPV positively correlated with SSI, and EDU positively correlated with SSN passively.
4. Street safety and quality of street green space promote older adults’ physical health by promoting VPA, and quality of GS and ACI promotes older adults’ physical health by promoting MPA and VPA.

The findings of this study add to the understanding of healthy older communities and contribute to the management of community settings to aid in policy development and implementation of healthy aging interventions. Our study provides valuable insights into optimizing elderly-friendly neighborhoods at both the local facility level and street space level.

## Figures and Tables

**Table 1:** Existing survey instruments.

Tools and Measures	Topics	Domains
Walking Route Audit Tool for Seniors (WRATS) [26]	Community street networks (the best walking routes for older adults, transportation, roads and streets, pedestrian facilities, bike facilities, traffic safety, parks and recreation, parks, recreation programs)	Community street networks
Neighborhood Environment Walkability Survey (NEWS) & Neighborhood Environment Walkability Survey-Abbreviated (NEWS-A) [27]	Community environment attributes relate to physical activity	Community environment
Measurement Instrument for Urban Design Quantities Related to Walkability [28]	Community environment (communities, architecture and building design, social and cultural environment)	Community environment
Measuring Urban Design Qualities—An Illustrated Field Manual [29]	Community street networks (imageability, enclosure, human scale, transparency, complexity)	Community environment
Instrumental activities of daily living (IADL) [22]	Instrumental activities (ability to use telephone, shopping, food preparation, housekeeping, laundry, mode of transportation, responsibility for own medications)	Daily activities (e.g., physical activities, social activities)
World Health Organization Quality of Life (WHOQOL)-BREF [30]	Multiple domains	Quality of life
Physical Activity Resource Assessment (PARA) Instrument [31]	Physical activity	Physical activity
Environmental Supports for Physical Activity Questionnaire [32]	Multiple domains	Multiple domains
Saint Louis Environment and Physical Activity Instrument [33]	Multiple domains	Multiple domains

**Table 2:** Contents of questionnaire.

Categories	Variables	Scale
Satisfaction of social infrastructure (SSI)		
Educational infrastructure (EDU)	1. The degree of subject satisfaction with each subtype of the infrastructure	5—Satisfied 4—Slightly satisfied 3—Neutral 2—Slightly unsatisfied 1—Very unsatisfied
Health and aged care services (HCS)		
Commercial services (CS)		
Arts and cultural infrastructure (ACI)		
Green and blue space (GBS) ^1^		
Recreational infrastructure (RI)		
Social infrastructure (SI)	2. The degree of subject satisfaction with the overall category of infrastructure.	
Satisfaction of street networks (SSN)		
Public communication space (PCS)	1. The degree of subject satisfaction with each subtype of the street networks	5—Satisfied 4—Slightly satisfied 3—Neutral 2—Slightly unsatisfied 1—Very unsatisfied
Green space (GS) ^2^		
Street accessibility (SA)		
Street walkability (SW)		
Diversity of rest facilities (DRF)		
Street networks (SN)	2. The degree of subject satisfaction with the overall category of infrastructure.	
Satisfaction of community environment (SCE)		
Community environment (CE)	The degree of subject satisfaction with the overall category of community environment.	5—Satisfied 4—Slightly satisfied 3—Neutral 2—Slightly unsatisfied 1—Very unsatisfied

^1^ GBSs represent community parks that provide recreational areas and certain activities and facilities for residents and help to enhance the beauty and environmental quality of neighborhoods and serve residents within a certain range of residential land; ^2^ GSs refers to the green space in roads and square land, including road green belts, traffic island green space, square green space and parking lot green space, and we added the details of GSs in the revised paper.

**Table 3:** Demographic information of participants.

Categories	Variables	Number of Participants	Percentage of Participants (%)
Community of Location	HUDONG	35	32.4%
	GUOYUAN	34	31.5%
	NANMEN	39	36.1%
Gender	Male	54	50
	Female	54	50
Education	Junior high school or below	30	27.8
	High school	60	55.6
	College or above	18	16.6
Household income (RMB/month)	Below 3000	23	21.3
	3000 to 5000	35	32.4
	5000 to 10,000	32	29.6
	10,000 and above	18	16.7
Household structure	Lives alone	28	25.9
	Lives with spouse	80	74.1
	Living with children	61	56.5
	Living without children	47	43.5

**Table 4:** Verification of indirect effects of SSI, SSN, and SCE through TPA.

Model	Path	Effect	SE	95%CI	
				Lower CI	Upper CI
SSI→IADL-score	Total indirect effect	0.111	0.075	0.009	0.285
SSN→IADL-score		0.094	0.066	0.002	0.251
SCE→IADL-score		0.068	0.046	0.004	0.175

**Table 5:** Verification of the indirect effect of SSI, SSN, and SCE through LPA, MPA, and VPA.

Model	Path			Effect	SE	95%CI	
						Lower CI	Upper CI
SSI→IADL-score	SSI	LPA	IADL-score	−0.069	0.112	−0.296	0.153
	SSI	MPA	IADL-score	0.111	0.094	−0.056	0.311
	SSI	VPA	IADL-score	0.234	0.067	0.118	0.376
	Total indirect effect			0.276	0.098	0.098	0.480
SSN→IADL-score	SSN	LPA	IADL-score	−0.136	0.090	−0.325	0.029
	SSN	MPA	IADL-score	0.146	0.085	0.014	0.342
	SSN	VPA	IADL-score	0.227	0.067	0.113	0.376
	Total indirect effect			0.237	0.093	0.074	0.438
SCE→IADL-score	SCE	LPA	IADL-score	0.192	0.233	−0.2550	0.682
	SCE	MPA	IADL-score	−0.131	0.207	−0.593	0.240
	SCE	VPA	IADL-score	0.251	0.117	0.034	0.492
	Total indirect effect			0.313	0.265	−0.245	0.799

**Table 6:** Results of the multilevel regression model for the SSI ^1^.

**Model**	**Model Summary**		**Variance** **Analysis**		**Unstandardized** **Coefficients**			
	**R**	**R^2^**	**F**	**Sig**	**B**	**Std. Error**	**t**	**Sig.**
(Constant)	0.845	0.714	58.638	0.000	3.058 ***	0.511	5.986	0.000
ACI ^2^					0.328 ***	0.059	5.585	0.000
GS ^3^					0.329 ***	0.063	5.248	0.000
EDU ^4^					−0.746 ***	0.135	−5.541	0.000
CS ^5^					0.170 *	0.072	2.356	0.021

* *p* < 0.05; *** *p* < 0.001. ^1^ Older adults’ satisfaction of community social infrastructure (SSI); ^2^ arts and cultural infrastructure (ACI); ^3^ green space (GS); ^4^ educational infrastructure (EDU); ^5^ commercial services (CS).

**Table 7:** Results of the multilevel regression model for the SSN ^1^.

**Model**	**Model Summary**		**Variance Analysis**		**Unstandardized** **Coefficients**			
	**R**	**R^2^**	**F**	**Sig**	**B**	**Std. Error**	**t**	**Sig.**
(Constant)	0.806	0.650	43.550	0.000	0.147	0.203	0.725	0.470
PCS ^2^					0.291 ***	0.072	4.056	0.000
DRF ^3^					0.235 **	0.068	3.461	0.001
SPV ^4^					0.230 **	0.068	3.387	0.001
GS ^5^					0.177 *	0.074	2.394	0.019

* *p* < 0.05; ** *p* < 0.01; *** *p* < 0.001. ^1^ Older adults’ satisfaction of community street networks (SSN); ^2^ public communication space (PCS); ^3^ diversity of rest facilities (DRF); ^4^ separation of people and vehicles (SPV); ^5^ green space (GS).

## Data Availability

Not applicable.
